# Endoscopic ultrasound-guided pancreatic pseudocyst drainage using a novel double-lumen dilator

**DOI:** 10.1055/a-2598-4058

**Published:** 2025-05-22

**Authors:** Takeshi Ogura, Jun Matsuno, Takafumi Kanadani, Ahmad Fikry Aboelezz, Hiroki Nishikawa

**Affiliations:** 1 38588 Endoscopy Center, Osaka Medical and Pharmaceutical University Hospital, Osaka, Japan; 2 2nd Department of Internal Medicine, Osaka Medical and Pharmaceutical University Hospital, Osaka, Japan; 3 68781 Department of Internal Medicine, Gastroenterology and Hepatology Unit, Tanta University, Tanta, Egypt

Endoscopic ultrasound-guided pancreatic pseudocyst drainage (EUS-PPD) is a well-established technique 1 2 3 . The procedure can be performed using a lumen-apposing metal stent (LAMS), allowing effective drainage because of the large diameter of the stent compared with plastic stents. However, a recent meta-analysis comparing EUS-PPD using plastic stents and LAMS reported similar technical and clinical success rates 4 . Additionally, plastic stent deployment still plays an important role from the perspective of cost-effectiveness in cases of pancreatic pseudocysts with fluid contents. However, the procedure requires deployment of two plastic stents, two guidewire insertions, and tract dilation after deployment of the first plastic stent. This leads to prolonged procedure time and higher costs. To overcome these issues, a novel dilation device (Meissa; Japan Lifeline, Tokyo, Japan) has been developed ( Fig. 1 ) 5 . The tip of this device is 2.3 Fr, and its maximum diameter is 7.4 Fr. In addition, a side hole is provided 2 cm from the tip. This enables contrast medium injection, aspiration of infected cyst fluid, and additional 0.025-inch guidewire insertion to be performed. Herein, we describe the technical procedure for EUS-PPD using this device.

**Fig. 1:**
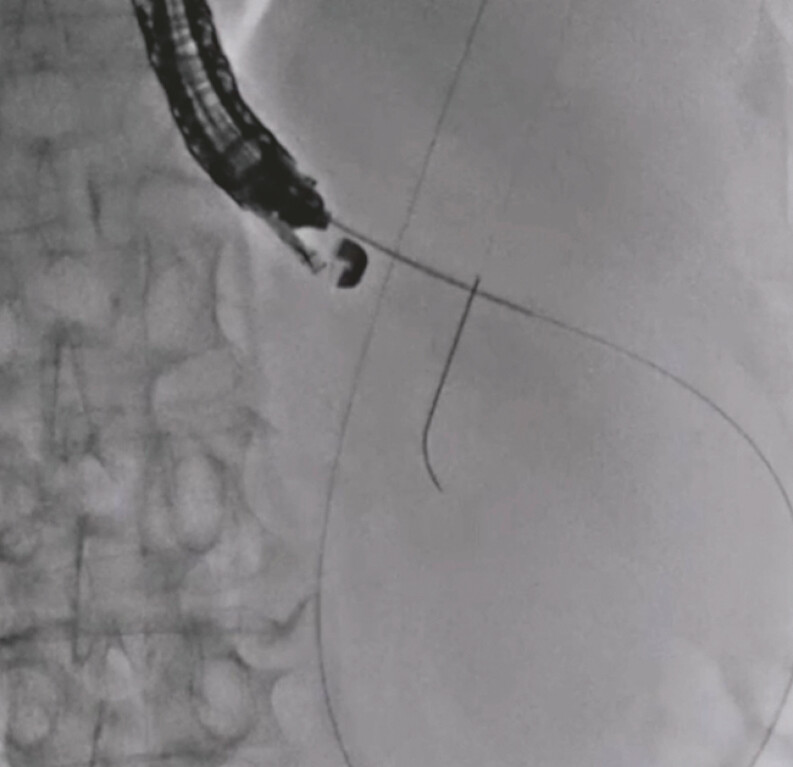
A novel dilation device (Meissa; Japan Lifeline, Tokyo, Japan).

A 55-year-old man was admitted to our hospital for the treatment of a pancreatic pseudocyst. Since EUS imaging showed that the pancreatic pseudocyst mainly contained fluid, EUS-PPD using a plastic stent was attempted. The pseudocyst was punctured using a 19G needle, and a 0.025-inch guidewire was inserted into the cyst ( Fig. 2 ). Next, a double-lumen dilator was smoothly inserted into the cyst. Then, an additional 0.025-inch guidewire was inserted into the cyst via the double-lumen dilator ( Fig. 3 ), and a 7-Fr double-pigtail plastic stent was subsequently deployed ( Fig. 4 ). Finally, insertion of an additional 7-Fr double-pigtail plastic stent was attempted. Since insertion of the double-lumen dilator had resulted in adequate tract dilation, this procedure was successfully performed without additional tract dilation or adverse events ( Fig. 5 , Video 1 ).

**Fig. 2:**
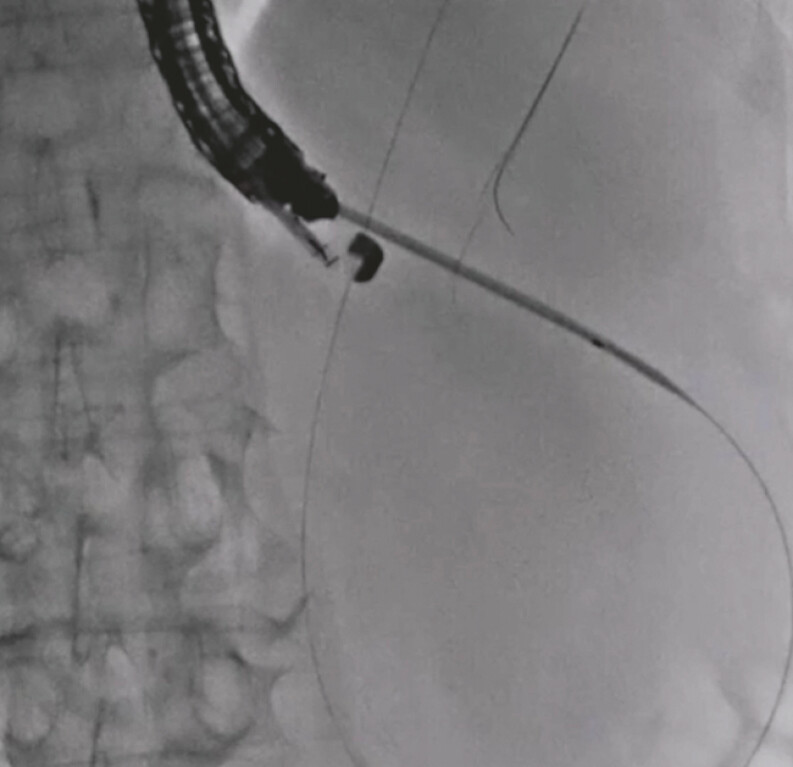
The pseudocyst is punctured using a 19G needle, and a 0.025-inch guidewire is inserted into the cyst.

**Fig. 3:**
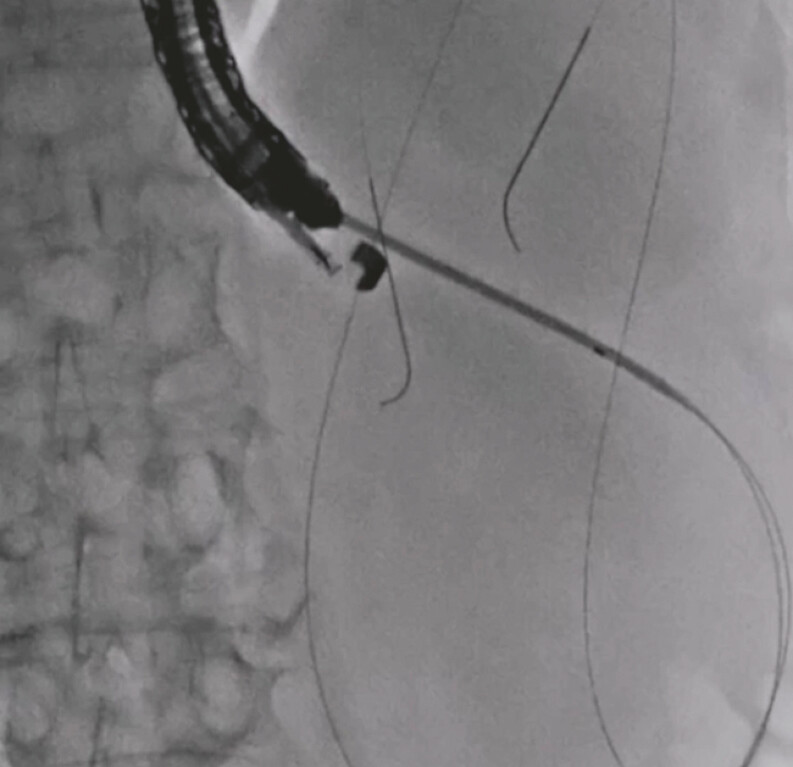
An additional 0.025-inch guidewire is inserted into the cyst via the double-lumen dilator.

**Fig. 4:**
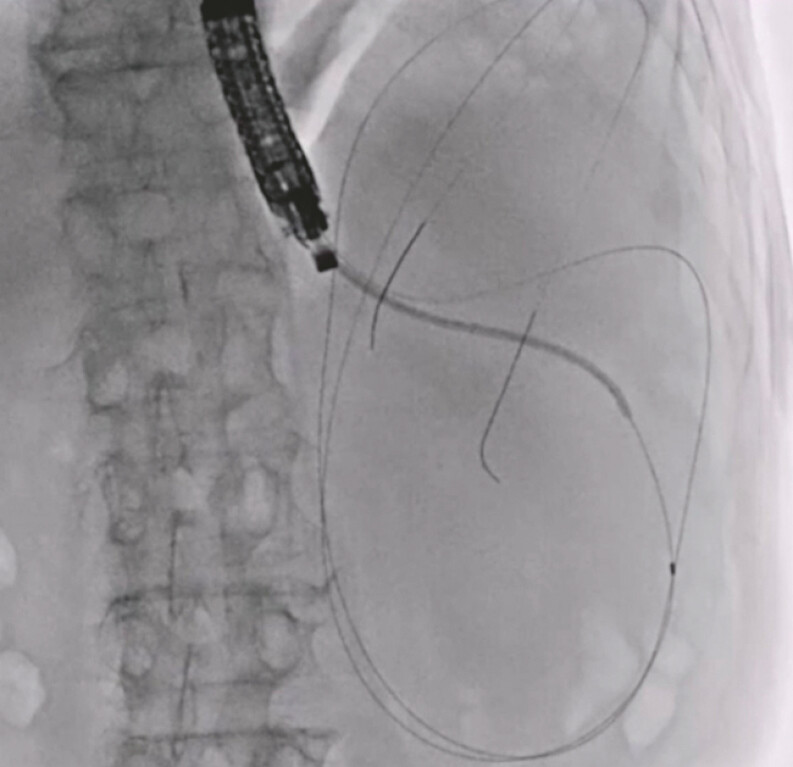
A 7-Fr double-pigtail plastic stent is subsequently deployed.

**Fig. 5:**
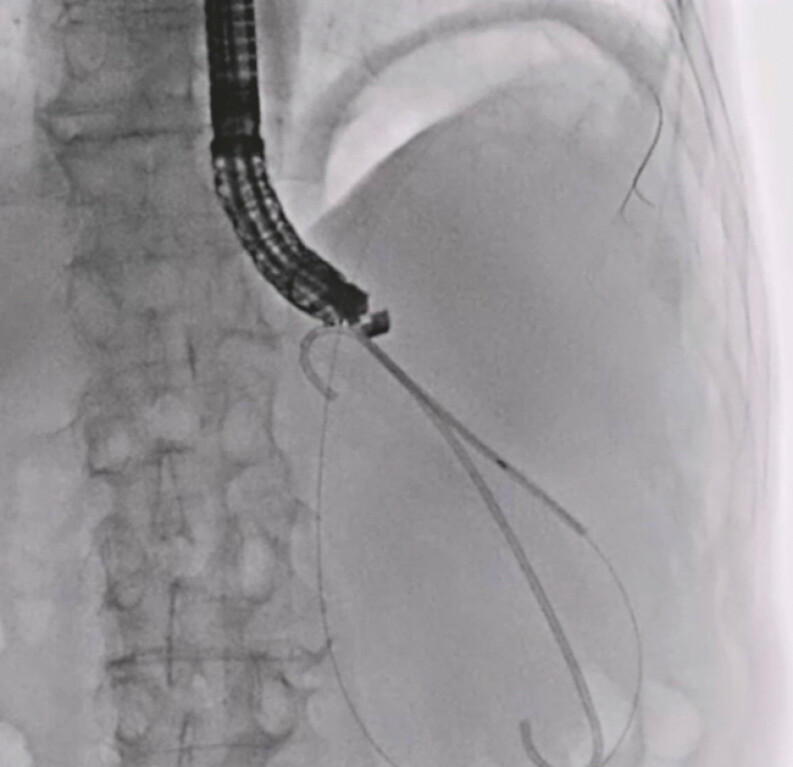
Insertion of an additional 7-Fr double-pigtail plastic stent is successfully performed without additional tract dilation.

Endoscopic ultrasound-guided pancreatic pseudocyst drainage using a novel double-lumen dilator.Video 1

In conclusion, the novel double-lumen dilator might serve a dual purpose during EUS-PPD, namely, allowing double guidewire deployment and tract dilation.

Endoscopy_UCTN_Code_TTT_1AS_2AJ

## Notes

Endoscopy E-Videos https://eref.thieme.de/e-videos
*E-Videos* is an open access online section of the journal *Endoscopy* , reporting on interesting cases and new techniques in gastroenterological endoscopy. All papers include a high-quality video and are published with a Creative Commons CC-BY license. Endoscopy E-Videos qualify for HINARI discounts and waivers and eligibility is automatically checked during the submission process. We grant 100% waivers to articles whose corresponding authors are based in Group A countries and 50% waivers to those who are based in Group B countries as classified by Research4Life (see: https://www.research4life.org/access/eligibility/ ). This section has its own submission website at https://mc.manuscriptcentral.com/e-videos .
